# From Pathogenesis to Treatment: Targeting Type-2 Inflammation in Eosinophilic Esophagitis

**DOI:** 10.3390/biom14091080

**Published:** 2024-08-28

**Authors:** Alberto Barchi, Francesco Vito Mandarino, Mona-Rita Yacoub, Luca Albarello, Luca Massimino, Edoardo Vincenzo Savarino, Federica Ungaro, Sandro Passaretti, Gwen M. C. Masclee, Silvio Danese, Albert J. Bredenoord, Edoardo Vespa

**Affiliations:** 1 Gastroenterology and Digestive Endoscopy, Motility Unit, IRCCS Ospedale San Raffaele, Via Olgettina 60, 20132 Milan, Italy; barchi.alberto@hsr.it (A.B.); mandarino.francesco@hsr.it (F.V.M.);; 2 Gastroenterology & Hepatology, Amsterdam University Medical Center, 1081 HV Amsterdam, The Netherlands; 3 Unit of Immunology, Rheumatology, Allergy and Rare Diseases, IRCCS San Raffaele Scientific Institute, 20132 Milan, Italy; 4 Pathology Unit, IRCCS San Raffaele Scientific Institute, 20132 Milan, Italy; albarello.luca@hsr.it; 5 Department of Surgery, Oncology, and Gastroenterology, University of Padua, 35128 Padua, Italy; 6 Gastroenterology Unit, Azienda Ospedale Università di Padova, 35128 Padua, Italy; 7 Faculty of Medicine, Università Vita-Salute San Raffaele, 20132 Milan, Italy

**Keywords:** eosinophilic esophagitis, type 2 inflammation, atopy, atopic march, EoE, biologics

## Abstract

Eosinophilic esophagitis (EoE) is a chronic inflammatory disorder of the esophagus. EoE shares a common pathogenetic mechanism with other chronic disorders pertaining to the type 2 inflammatory spectrum, such as atopic dermatitis (AD), allergic rhinitis (AR), asthma, and chronic rhinosinusitis with nasal polyps (CRSwNP). The recent advancements in EoE pathogenesis understanding have unveiled new molecular targets implied within the “atopic march” picture as well as specific to EoE. These discoveries have led to the clinical evaluation of several novel drugs (monoclonal antibodies and immune modulators), specifically aimed at the modulation of Th2 inflammation. In this comprehensive review, we have focused on the subtle mechanisms of type 2 inflammatory disorders, highlighting the similarities and differences with EoE, taking a deeper look into the evolving field of biologic therapies, already approved or under current investigation.

## 1. Introduction

Eosinophilic esophagitis (EoE) is an esophageal inflammatory chronic disease characterized by an immune-mediated profile [1]. EoE is characterized by the T helper 2 (Th2) inflammatory pathway, driving the eosinophilic infiltration of the esophageal wall, leading to obstructive symptoms, mainly dysphagia and food impaction [2]. EoE is characterized by a prevalence (up to 40.4 cases per 100,000 inhabitants) and incidence (5.31 cases per 100,000 inhabitant) that are constantly increasing [3]. EoE predominantly affects the male gender, with a male/female ratio of 3:1 [4], and there is an increased relative risk among siblings, particularly monozygotic twins, suggesting a subtle hereditary component [5]. The downside of the chronicity of the inflammatory insult is the progressive remodeling of the esophageal wall, leading to fibrosis deposition and narrowing [6]. Esophageal remodeling is burdened by an increased risk of food impaction and stricture formation, requiring endoscopic dilation [7]. According to the most recent guidelines [8,9], EoE diagnosis is still provided by the presence of >15 eosinophils per High-Power Field (HPF) in at least one esophageal location in the presence of obstructive symptoms. The endoscopic appearance can be pathognomonic (white exudates, linear furrows or rings), but still, a percentage of endoscopic examinations may be negative [10]. Pathology examination is pivotal to characterizing EoE, going beyond eosinophil count, addressing epithelial and sub-mucosal alterations, such as dilated intercellular spaces (DISs), basal zone hyperplasia (BZH), or surface epithelial alterations (SEAs), all comprising a recently developed [11] and validated histologic score [12]. For a long time, EoE treatment has been based on the use of proton pump inhibitors (PPIs) at various dosages, based on their demonstrated role in inhibiting eosinophil mediator activation [13]. Swallowed topical corticosteroids (STCs) have been borrowed from the allergology setting for their effectiveness in silencing the inflammatory cascade in EoE but have mostly been used off-label [14]. Recently, the development of a novel STC, with a specific formulation designed for esophageal delivery, has revolutionized EoE treatment, providing optimal treatment efficacy with low side effects [15]. Nonetheless, PPIs remain the first-line treatment alongside STCs, displaying still decent rates of histological and clinical responses and rare adverse events [16]. Dietary therapies continue to play a role in managing EoE mostly in selected patients eager to be compliant to such restrictive dietary regimens [2]. The advent of biological therapies, addressing complex cases refractory to STCs, have shed new light on the importance of understanding EoE pathogenesis [16]. On the one hand, biologics have shown optimal histologic remission rates, proving efficiency in targeting type 2 pathways, but on the other hand, low clinical success rates suggest that eosinophils might be the wrong target [17]. This narrative review has the aim of overviewing the current literature available on EoE pathogenesis, the shared environment with other type 2 disorders, and the recent advancements in type 2-targeted treatments.

## 2. Type 2 Inflammation: “Atopic March”—Common Pathogenesis between Atopic Dermatitis (AD), Asthma, Allergic Rhinitis (AR), Chronic Rhinosinusitis with Nasal Polyps (CRSwNP), and EoE

**Figure 1:**
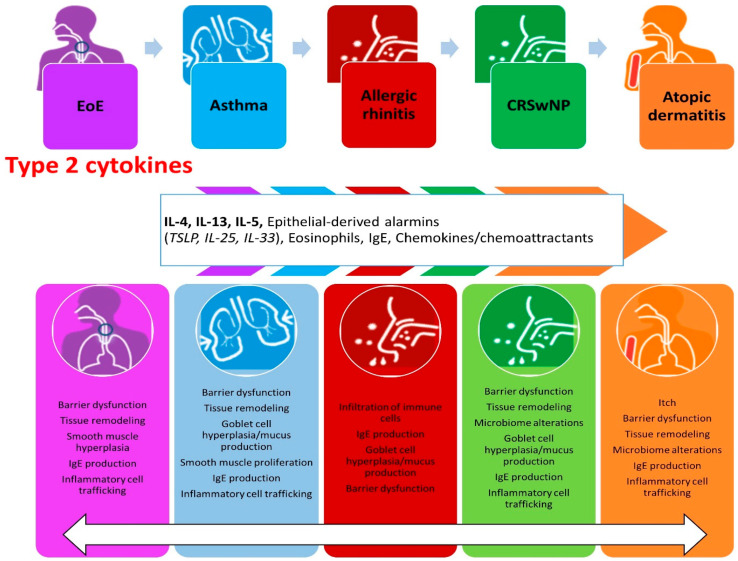
Principal disorders related to type 2 inflammation with pivotal molecules implicated in Th2 immune response and leading biological mechanisms.

Type 2 inflammatory disorders are a spectrum of immune-mediated diseases, sharing a common pathogenesis [18]. The common pathways within these disorders could be encompassed by the term “atopic march” [19]. From epidemiological studies, AD presents in 15–25% of children [20], which usually overlaps with asthma for 20% (up to 60% in severe AD cases) [21]. The co-presentation of asthma and AR is even more relevant (up to 80%) [22]. AD is a chronic skin disorder characterized by itching, skin inflammation, and epidermal barrier dysfunction [23]. AD is usually considered to be the first red alert leading the “atopic march”, frequently predisposing patients to the other type 2 inflammatory conditions [23]. Asthma involves the respiratory system, causing airflow trapping and chronic obstruction, in which the disruption of the bronchial epithelium plays a pivotal role [24]. The correlation between CRSwNP and the “atopic march” is much more complex to describe. CRSwNP is a disorder characterized by rhinorrhea and nasal obstruction with radiologically detectable signs [25]. Even though CRSwNP did not show a clear overlap, or a distinct progression from AR (up to 5% of AR prevalence, similar to general population [26]), an allergic diathesis has been reported in more than 85% of CRSwNP patients [27]. Moreover, biologic therapies active for other type 2 disorders have been found to be effective in CRSwNP patients [28]. EoE’s relation to atopic diseases has been extensively exploited [29]. Large epidemiological studies have demonstrated a significantly higher rate of atopic comorbidities in EoE patients compared to the general population [30]. Furthermore, it has been reported that the higher the number of atopic comorbidities, the higher the risk of developing EoE, potentially classifying it as a late protagonist of the “atopic march” [31]. Epithelial (EC) and dendritic cell (DC) crosstalk is the background of the type 2 immune response [32]. Epithelial “alarmins”, mainly Interleukin (IL)-25, IL-33, and the thymic stromal lymphopoietin (TSLP), are a group of epithelial cytokines released in the first steps of the inflammatory cascade, after an antigenic stimulus [32]. The antigens activating the type 2 cascade are diverse, ranging from multicellular organisms and bacterial wall components to food and aeroallergens [33]. The innate immune system, through DCs, type 2 innate lymphoid cells (ILC2s), and basophils, is a key factor in the initiation of the immune cascade, contributing to the production of “alarmins” [34]. DCs stimulate T naïve lymphocytes, resident in lymph nodes, to differentiate into Th2 cells, thanks to the aid of basophil-derived IL-4, which is continuously supplied by the activation of ILC2s [35] (Figure 1).

While IL-4 can be considered as the booster of the innate type 2 immune response, IL-13 is definitely to be regarded as the protagonist of the adaptive system [36]. Highly specialized central-memory effector Th2 cells, expressing CD4 and characterized as chemoattractant receptor-homologous molecule (CRTH2)-positive, are crucial in producing huge loads of IL-13, together with activated ILC2s [37]. IL-13 plays a determinant role in eosinophil chemotaxis, goblet cell hyperplasia, smooth muscle contractility, and collagen deposition [38]. Together with IL-13, IL-5 is the main driver for eosinophil differentiation in the bone marrow, while IL-9 has an important role in ILC2 and mast cell (MC) activation [32]. Even immunoglobulins (Igs) are involved in the pathogenesis of Th2 disorders. As in the allergic reaction pathway, effector Th2 cell polarization and the increased production of IL-4 stimulate B cells to activate and increase IgE production [39]. This mechanism has been therapeutically targeted by the use of Oral Immuno-Therapy (OIT) in IgE-mediated food allergies, which have been described as part of the atopic spectrum [40]. MCs, activated by the prominent stimulus IL-9 produced by effector Th2 cells and ILC2s, express several surface receptors, being *FcεRi*, c-KIT, and the sialic acid-binding immunoglobulin-like lectin (Siglec) family, the most relevant and all potential treatment targets [41]. Altered barrier function is one of the fundamental building blocks of type 2 pathogenesis [32]. An early deficiency in Filaggrin (FLG) expression, mainly due to the genetic mutation of FLG-related genes, seems to be the initial driver of skin barrier disruption in AD, reducing the defensive mechanisms against antigens and allergens [42]. In asthma, a deficiency in epithelial tight junctions (predominantly a reduced E-cadherin, alpha-catenin, zonulin-1, and occludin expression) has been described [43]. The increased barrier permeability paves the way for EC activation and the production of both “alarmins” and danger-associated molecular patterns (DAMPs), stimulating DCs for antigen presentation via Toll-Like Receptor (TLR) stimuli, initiating the type 2 inflammatory drive [44]. From earlier studies, it seems likely that an early exposure to certain substances (i.e., cigarette smoking) or pathogens (Respiratory Syncytial Virus [RSV]) could increase the expression of TLR4 on ECs, leading to a vulnerability to allergic responses [45]. The type 2 response, with an increased production of Th2-related cytokines (IL-4, Il-5, and IL-13), fuels this vicious cycle, further hindering barrier permeability [46]. Many hypotheses have tried to unravel the common pathway shared by type 2 inflammatory disorders. The “exposome” theory links the epithelial barrier disruption and allergic vulnerability to the exposure to environmental harmful substances (detergents, microplastics, nanoparticles, food additives, and many more) [47]. The composition of the bacterial environment of the skin has been addressed as a potential factor in the “atopic march”, clarified by the demonstrated role of the increased abundance of *S. aureus* as a trigger of AD, most likely through Pathogen-Associated Molecular Patterns (PAMPs) stimuli [48]. Furthermore, in recent years, the importance of the gut microbiota and its alteration in a dysbiotic manner have been emphasized [49]. The genetic predisposition is nonetheless the most relevant topic in type 2 immune-mediated disorders. Marenholz and colleagues recently performed a Genome-Wide Association Study (GWAS) meta-analysis in asthma and AD patients, identifying two genome-wide significant single nucleotide polymorphisms (SNPs) in the epidermal differentiation complex (EDC), associated with FLG gene [50]. Another meta-analysis brought new light upon the epigenetic regulation of the atopic predisposition, evaluating new DNA methylation patterns [51]. In this atopic picture, EoE is believed to be a late protagonist in susceptible individuals. 

## 3. Type 2 Inflammation Is Key in EoE Pathogenesis: Review of Molecular Mechanisms

EoE pathogenesis has been intensively studied in recent years. EoE has an underlying genetic susceptibility, widely proven [1]. Blanchard et al. identified a section of the human genome, termed the “EoE transcriptome”, with a conserved expression of multiple gene loci, in the esophagus of EoE patients [52]. Among the conservatively expressed genes, CCL26, encoding for eotaxin 3 (an eosinophil chemoattractant and activator), resulted to be the most correlated among other gene loci comprising the EDC, a transcriptional “hot spot” in the atopic diseases’ genetic drive [53]. One of the largest GWASs revealed how the Calpain 14 (CAPN14) gene, encoding for an IL-13-induced cysteine protease, resulted to be the highest correlated gene with active EoE [54]. Furthermore, based on previously confirmed risk loci for allergic sensitization [55], the authors found a strong association of some genes implied in Th2 allergic responses in EoE compared to healthy individuals (particularly, TSLP, LRRC32, and LPP) [54]. Even though TSLP could act as an alarmin to activate ILC2s and effector Th2 cells as in the other atopic diseases, CAPN14 is believed to be tissue-specific for EoE [56]. These genetic insights reflect the proven heritability pattern of the disease. Several studies have confirmed the increased risk (up to 64-fold) in EoE brothers, with a monozygotic twin’s concordance of 58% [5]. The genetic predisposition of EoE underlies the atopic/allergic substrate. The exposure to aero- or food antigens is pivotal in initiating the Th2 response [57]. Moreover, seasonal allergies seemingly play a role in the pathogenesis [58]. Studies on elimination diets have enlightened this pathway with clinical applications [59]. The first proof of the crucial role of the Th2 immune response and related cytokines in EoE pathogenesis came from animal studies. Akei et al. injected wild-type mice with epicutaneous antigens (ovalbumin or *Aspergillus fumigatus*), evidencing the role of IL-5 (via STAT5) in eosinophil production and recruitment with a determinant contribution by IL-4 and IL-13 via STAT6 [57]. The relevance of IL-5 in EoE has been widely re-estimated after the incomplete reduction in esophageal eosinophilia and the inefficacy in the clinical activity of IL-5-targeted monoclonal antibodies (mAbs) [60]. Blanchard and colleagues further explored the role of IL-13 in EoE, documenting the induction of a transcription profile overlapping with EoE patients in esophageal epithelial cells treated with the injection of IL-13 [53]. Zuo et al. demonstrated the role of IL-13 in inducing esophageal remodeling in transfected mice expressing the IL-13 gene in the lung, via an eotaxin 1-dependent pathway [38]. Moreover, the authors evidenced the inhibitory effect of the IL-13α2 receptor [38]. Eotaxins are determinants for eosinophil recruitment, as demonstrated by earlier studies on eotaxin-deficient mice [61]. The absence of eotaxin production is characterized by a reduced eosinophil accumulation in GI tissues [61]. Among eotaxins, eotaxin 3, encoded by the abovementioned CCL26 gene, appears to be prominently stimulated by IL-13 [53]. IL-13 (via STAT6) also contributes to eosinophilic chemotaxis, goblet cell hyperplasia, and smooth muscle contractility and terminally to collagen apposition and esophageal remodeling [38]. The overexpression of IL-13 in transgenic mice with the stimulation of the Th2 immune response via doxycycline administration caused significant epithelial thickness, with augmented esophageal epithelial proliferation, increased angiogenesis, and collagen deposition [38]. Esophageal remodeling seems to be highly dependent on the IL-13 induction of Transforming Growth Factor (TGFβ), capable of determining periostin induction, smooth muscle contraction, collagen deposition, and myofibroblast differentiation (via SMAD signaling) [38]. Furthermore, IL-13 has been related to epithelial barrier disruption. Davis and colleagues demonstrated that the epithelial loss of permeability in EoE follows an IL-13-induced CAPN14-dependent pathway [62]. CAPN14 seems to induce Desmoglein-1 (DSG-1) and/or Desmoplakin (DSP) downregulation, leading to loose intercellular spaces [62]. Human Igs have been extensively investigated in EoE. The IgE class, historically related to allergic disorders, proved not to be a trigger for EoE, while several works recently highlighted the possible role of tissue-resident IgG4s [63]. IgG4s related to specific food antigens have been found in blood and esophageal samples of EoE patients, and their decrease after Food Elimination Diets (FEDs) has been described [64]. Th2 cytokine production in EoE is guided by ILC2s and effector Th2 cells expressing the CRTH2 receptor, which has been addressed by targeted treatments [65]. These cells are activated via the exclusive pathway involving the already-mentioned “alarmins”, TSLP, IL-25, and IL-33 [66]. MCs are increasingly being studied in EoE pathogenesis, thanks to their capacity of inducing smooth muscle hypertrophy [67], producing Th2 cytokines like IL-9 and IL-13 [67], and their presumable role in esophageal remodeling via TGF-β induction [68]. Even eosinophil degranulation products, such as eosinophil-derived neurotoxin (EDN), eosinophil peroxidase (EPO), eosinophil cationic protein (ECP), or major basic protein (MBP), are possible contributors in EoE development, via DC activation and membrane permeability induction [69]. Studies on their role as potential non-invasive biomarkers are ongoing. Less is known about the impact of the esophageal microbial environment on EoE. Recent studies demonstrated a potential role of EoE dysbiosis, particularly at the level of the bacteriome, fungiome, and virome, in discriminating between EoE, GERD, and healthy subjects [70]. This field of research is advancing but still lacks therapeutic targets [49].

## 4. Therapies Targeting Type 2 Inflammation: Evidence from Trials with Available Therapies and How They Target T2 Inflammation in EoE

**Figure 2:**
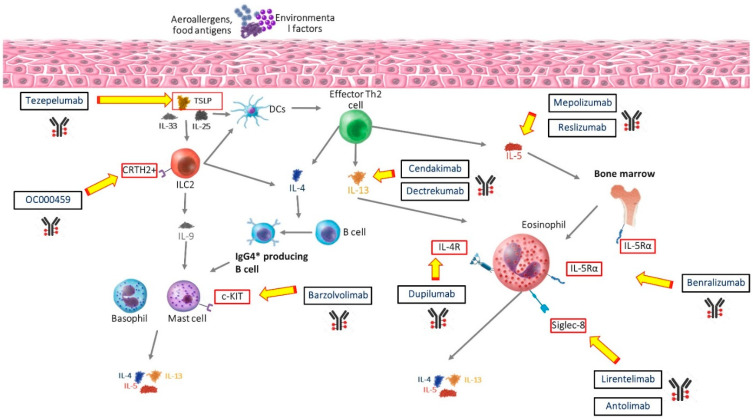
Molecular pathways within eosinophilic esophagitis (EoE) pathogenesis, displaying the most relevant molecular targets of novel biologic drugs addressing type 2 inflammatory drive.

The main goal of current EoE treatments is to achieve histologic remission. While this is an evolving concept, as esophageal eosinophilic depletion does not systematically translate into clinical efficacy (discussion below), the widely accepted definition is <15 eosinophils per HPF on esophageal biopsies [8]. PPIs are still used off-label in EoE patients, often naïve to other treatments, thanks to their safe profile and still quite acceptable short-term efficacy [16]. Usually, after the failure of an induction therapy with PPIs, steroids come to the rescue. A novel orally dispersible formulation of budesonide compounds has revolutionized the picture of EoE treatment [71], replacing aero-dispersible swallowed topical corticosteroids (STCs) and reaching optimal levels of efficacy [16]. Nonetheless, the existence of steroid-refractory patients and the urge to explore the apparent clinical–histological disconnection, found in several clinical trials, led to experimental biological therapies to address the common pathogenetic Th2-related targets (Table 1) [17]. The main mechanisms of action of the currently available (or under investigation) biological drugs in EoE are displayed in Figure 2.

### 4.1. Interleukin-4

Dupilumab is a recombinant fully human monoclonal IgG4 antibody directed against the type II IL-4 receptor alpha subunit (IL-4Rα) [72]. The IL-4 receptor harbors the IL-4Rα and IL-13Rα subunits and can be found in non-hematopoietic cells [72]. Before being investigated in EoE, dupilumab received Food and Drug Administration (FDA) and European Medicines Agency (EMA) approval for other Th2-related diseases, namely AD [73,74], asthma [75,76], and CRSwNP [77]. For EoE, dupilumab was firstly investigated in a phase II Randomized Controlled Trial (RCT) on 23 EoE patients, reaching a significant symptomatic response (*p* < 0.031), histologic remission (*p* < 0.0001), and endoscopic improvement (*p* < 0.0006) at 12 weeks compared to the placebo [78]. These results were later confirmed in the long-term extension (up to 52 weeks) phase III RCT on 203 total patients [79]. This RCT was designed as a two-part trial, with part B investigating two different dupilumab dosages (300 mg s.c. once per week and once every 2 weeks), without meeting the co-primary endpoint of clinical response [79]. Recently, the long-term results of these two cohorts were published, reporting a maintained histological, endoscopic, and clinical improvement up to 52 weeks of dupilumab once per week compared to the reduced dosage [80]. A just recently published RCT on pediatric EoE patients (age range 1–11 years) has shown 68% and 58% histologic remission after 36 weeks of induction with a higher and lower dose of dupilumab, respectively [81]. Significant differences (*p* < 0.001) compared to the placebo were reported also for the endoscopic and clinical endpoints. Notably, these outcomes were maintained in the long term (up to 52 weeks) for both dose regimens [81]. Dupilumab is being investigated in relation to food reintroduction regimens in an ongoing monocentric trial (NCT05247866), recruiting patients between 6 and 25 years of age, and in a large multicentric international trial (the REMODEL trial) (NCT06101095) investigating its effect on esophageal remodeling. Dupilumab is the only biologic compound currently FDA- and EMA-approved for the treatment of EoE (>12 years) worldwide. 

### 4.2. Interleukin-5

As the major trigger for eosinophil production and migration, IL-5 represents a potential natural target of EoE treatment. Benralizumab is a fully humanized, afucosylated mAb against the alpha subunit of the IL-5 receptor (IL5Rα), which can be found on the surface of eosinophils and in the bone marrow [82]. Benralizumab has shown great efficacy in asthma in several phase III RCTs [83,84]. It has been evaluated in the “MESSINA” trial, a large phase III RCT, enrolling EoE patients between 12 and 65 years of age (NCT04543409). The study was terminated early after interim analyses showed a failure in meeting the clinical co-primary endpoint. The results of this trial have recently been provided, confirming a relevant histologic efficacy of benralizumab compared to the placebo (80.8 percentage points, *p* < 0.001) but no improvement in the clinical and endoscopic outcomes (*p* = 0.18) [85]. This result clearly poses the issue of going beyond eosinophil-directed therapies, as confirmed by a published RCT on the same drug in Eosinophilic Gastritis (EG) [86]. Reslizumab and mepolizumab are two fully humanized IgG1k mAbs, with high affinity for IL-5, preventing its binding to IL-5rα [87,88]. Both drugs have been approved for the treatment of eosinophilic asthma, with good efficacy and safety [89,90]. Reslizumab has been evaluated in one RCT with three arms of increasing dosages compared to a placebo, in 169 total EoE patients [91]. Even though a significant (*p* < 0.001) reduction in eosinophil count among all treatment groups was registered, no significant clinical improvement was met [91]. Mepolizumab has been investigated in two RCTs, one phase I placebo-controlled, evidencing a significant reduction in esophageal eosinophils compared to the placebo (*p* < 0.03) together with EoE-related transcripts [92], and in a phase II RCT with two arms of different dosages, showing no differences between them [93]. Recently, the results from a large phase III placebo-controlled RCT showed a histologic improvement but confirmed the lack of clinical response [60] (Table 1). 

### 4.3. Interleukin-13 (IL-13) 

IL-13 is one of the major targets of the ongoing biologic revolution in EoE. IL-13 has the ability to bind two different subunits: IL-13Rα1, already described, which binds IL-13 with low affinity only coupled with the dimeric IL-4Rα [94], and IL-13Rα2, with a high affinity for IL-13, negatively regulating IL-13Rα1-IL-4Rα binding and driving fibrosis via macrophage interaction [95]. The first pathway has been extensively addressed with dupilumab. Among several mAbs targeting the second pathway and never clinically investigated (like tralokinumab [96]), cendakimab (RPC4046 or *CC-93538*) has shown promising results. It prevents the binding of IL-13 to both alpha subunits and has reported high levels of remission in a phase II trial [97]. The improvements in the endoscopic, histologic, and symptomatic outcomes have been proven stable in a recently published long-term extension trial (up to 52 weeks) [98]. A large phase III trial in the US is actively recruiting adult EoE patients [NCT04991935]. Another anti-IL-13 mAb (Dectrekumab or *QAX576*) has demonstrated improvement in the esophageal eosinophil count and in the EoE-related transcription profile expression in a small placebo-controlled RCT on 17 EoE patients [99].

### 4.4. TSLP, Sialic Acid-Binding Immunoglobulin-like Lectin 8 (Siglec-8), and CRTH2 Receptor

TSLP is a key actor in Th2-related inflammation and has been addressed in a large RCT in moderate-to-severe asthma, reporting reduced re-exacerbations compared to the placebo [100]. An EoE phase III RCT is actively recruiting patients (NCT05583227). Siglec 8 is a member of the Siglec family of MC and eosinophil surface receptors, which plays an inhibitory role on CD33 substrates [101]. The binding of Siglec 8 with a mAbs induces the apoptosis of eosinophils and decreases MC activation [102]. Antolimab and lirentelimab are the two available anti-Siglec 8 antibodies (AK002), which have been evaluated in EG and Eosinophilic Duodenitis (ED) in 43 EG/ED patients, showing a significant reduction both in tissue eosinophil concentrations (*p* < 0.001) and symptoms (*p* < 0.004) compared to the placebo [103]. Lirentelimab is under investigation also in chronic urticaria (NCT03436797) and in AD (NCT05155085). Given its beneficial effects, lirentelimab is currently under investigation also in EoE patients in a phase II/III placebo-controlled randomized trial (KRYPTOS trial), which is enrolling EoE patients > 12 years old (NCT04322708). The preliminary results of this trial have been published in abstract form, reporting having met the histologic but not the clinical endpoint (*p* = 0.23) [104]. CRTH2 is a G-protein-coupled receptor expressed by effector Th2 cells but also by eosinophils, basophils, and ILC2s [105]. Its ligand is prostaglandin D2, and when bonded, it favors the recruitment of eosinophils and basophils, inducing Th2 cytokine production [106]. OC000459 is an indole acetic acid derivative with selective antagonism of CRTH2 [107]. It has been evaluated in persistent asthma [108,109] and in patients with a pollen allergy [110], with good outcomes. Straumann and colleagues, in a phase II placebo-controlled RCT on 26 adult EoE patients, reported a moderate histologic (*p* < 0.025) and clinical response (*p* < 0.035) [65]. 

### 4.5. Other Molecular Targets 

The role of MCs in EoE is currently taking the spotlight [111]. Single-cell RNA sequencing studies have proven the presence of MC subtypes, which can produce high levels of IL-13, proliferate, and degranulate during active inflammation [112]. The EoE transcriptome has shown the expression of MC-related genes including c-KIT, which is the target of a novel mAb (barzolvolimab) inhibiting the KIT pathway (NCT05774184). A phase II trial on this drug is currently in the recruiting phase (NCT05774184). IL-15 is an inflammatory cytokine which stimulates the differentiation of T cells and their activation in the absence of antigen presentation [113]. IL-15 also has the role of activating and maintaining Natural Killer (NK) cells, with a likely role in inducing EoE [114]. IL-15 expression was reported to be increased in EoE and lowered after treatment response [115]. Currently, a phase I trial with CALY002, a novel anti-IL-15 mAb, has just finished the recruiting phase in a cohort of celiac disease and EoE patients (NCT04593251). The preliminary results seem promising [116]. Concerning the allergic IgE-mediated pathway in EoE, IgEs have been studied as potential candidate targets in EoE [117]. Food allergies have been shown to be central in the development of the disease, thanks to empirical data on elemental diets drastically improving the main features of EoE [118]. For this reason, a phase II RCT was conducted, assessing the efficacy and safety of the anti-IgE drug omalizumab, without reporting significant histologic or clinical improvement [63]. Given early discoveries pointing out a potential upregulation of Tumor Necrosis Factor alpha (TNFα) in EoE [52], Straumann and colleagues designed an open-label 4-week prospective trial on infliximab (a chimeric IgG1 mAb), not evidencing an improvement in histological or clinical parameters in the first three enrolled patients [119]. Several other immune-modulating patterns are currently under investigation. A novel molecule, IRL201104, a small peptide fragment of Chaperonin 60.1, able to inhibit leukocyte trafficking and infiltration in tissues, is currently in the recruiting phase in a randomized trial on adult EoE patients (NCT05084963). Another phase II RCT (VOYAGE trial) (NCT04682639) is ongoing on etrasimod, a S1P-receptor modulator, which has shown effectiveness in the IBD field [120]. The preliminary results of this RCT showed a clinical and histological improvement versus the placebo in the first 24 weeks of induction with two different dosages, maintaining a sustained response throughout week 52 [121].

## 5. Future Directions

Type 2 disorders are a group of immune-mediated disorders pertaining to the wide “atopic” spectrum, AD usually being the first manifestation in children. The initial trigger is usually the exposure to food or aeroallergens, causing the release of epithelial alarmins and type 2-specific cytokines. This inflammatory drive activates several cell types, including eosinophils, DCs, effector Th2 cells, MCs, and basophils. EoE is believed to be the last step of the “atopic march”, sharing several features with other Th2 disorders like AD, AR, asthma, and CRSwNP. Genomic and transcriptomic studies have unveiled a genetic signature underlying EoE, with the expression of genes implied in chronic inflammation and epithelial barrier disruption. Eosinophils have been elected as the main target in EoE management, with histological remission being related to disease control and reduced relapse risk. However, an increasing amount of evidence raised the dilemma of how even strict histological control may not be the optimal endpoint. The failure in meeting clinical and endoscopic endpoints of the most recent large RCTs on biologic drugs active directly towards eosinophil activation (benralizumab and mepolizumab) clearly advocates the exploration of new therapeutic avenues. IL-13 seemed the most peculiar pathway to be addressed in EoE targeted treatment, being specific and related not only to active inflammation but also to chronic remodeling. The great enthusiasm around dupilumab in atopic disorders (targeting the IL-13 pathway via IL4 dimeric receptor binding) has given rise to large, randomized trials clearly stating its superiority to the placebo in inducing histological clinical remission. Furthermore, the effect of dupilumab on the transcriptomic profile of EoE has highlighted its usefulness in the advanced stages of the disease. All these efforts led the way to its approval as the first mAb in EoE. On the same side, data on cendakimab, already evaluated in large phase III trials, seem equally reassuring. Concerning the targeting of other EoE-specific targets, an anti-TSLP drug (tezepelumab) is under the magnifying glass, with reassuring interim results. Exploring the underlying connections between EoE and the other Th2 disorders, particularly the interesting role of different cell types, like ILC2s, MCs, and basophils and their interaction with the atopic spectrum, seems promising. The results on anti-cKIT mAb (barzolvolimab) are eagerly awaited, while a preliminarily anti-siglec-8 antibody (lirentelimab) appeared effective in treating EoE inflammation. In the same way, new immune-modulator drugs (etrasimod, OC000459, or CALY002), already effective in other settings, are currently under investigation with optimistic preliminary results. The current picture of EoE treatments is constantly evolving, distancing from a mere anti-inflammatory and anti-eosinophil purpose (already effectively acquired with novel formulations of STCs available) to approach the Th2 immune environment. Three main gaps are to be addressed by research efforts: (a) to unravel the subtle molecular mechanisms underlying the Th2-prevalent EoE phenotype, distinguishing it from a GERD-related EoE phenotype; (b) to reveal the molecular targets in advanced fibrotic EoE in an attempt to reverse esophageal remodeling and obtain “restitutio ad integrum”; and (c) to find new non-invasive biomarkers for a cost-effective and faster diagnosis and to prevent chronic relapse. 

## Figures and Tables

**Table 1:** Molecular targets in EoE advanced treatments: rationale and current evidence.

Target	Rationale	Trials	NCTs	Drug	Pharmacodynamics	Approved
IL-4	Via STAT6 pathway Th2 cell differentiation MC and basophil activation B cell differentiation (IgE/IgG4 production) Eosinophilic recruitment	5	→ NCT02379052 (2015) # → NCT03633617 (2018) # → NCT04394351 (2020) # → NCT05247866 (2022) * → NCT06101095 (2023) ^	Dupilumab (5)	Anti IL-4Rα mAb	Yes (>12 years)
IL-13	Via STAT6 pathway Eotaxin production Calpain 14 induction DSG/DSP downregulation Smooth muscle hypertrophy Eosinophil chemotaxis Collagen deposition (remodeling)	4	→ NCT01022970 (2009) # → NCT02098473 (2014) # → NCT04991935 (2021) ^	Dectrekumab (1) Cendakimab (2)	IL-13Rα1–2 mAb	No
IL-5	Via STAT5 pathway Eosinophil production in bone marrow Eosinophil recruitment and activation	6	→ NCT00274703 (2005) # → NCT00358449 (2006) # → NCT00538434 (2008) # → NCT00635089 (2008) # → NCT03656380 (2019) # → NCT04543409 (2020) #$	Reslizumab (2) Mepolizumab (3) Benralizumab (1)	IL5Rα mAb (benralizumab) Anti-IL5 mAb (mepolizumab, reslizumab)	No
TSLP	Major epithelial “alarmin” Promoting Th2 differentiation Associated with multiple allergic disorders	1	→ NCT05583227 (2022) ^	Tezepelumab (1)	Anti-TSLP mAb	No
Siglec-8	Receptor on MCs and eosinophils Eosinophil apoptosis MC reduced activation	1	→ NCT04322708 (2020) ^	Lirentelimab (1)	Anti-Siglec-8 mAb	No
CRTH2	G-protein-coupled receptor expressed by effector Th2 cells, basophils, and ILC2s	1	→ NCT01056783 (2010) #	OC000459 (1)	Selective antagonist of CRTH2 receptor	No
c-KIT	MC-related gene upregulated in EoE transcriptome	1	→ NCT05774184 (2023) ^	Barzolvolimab (1)	MCs anti-c-KIT mAb	No
IL-15	Th2 cell differentiation without antigen presentation	1	→ NCT04593251 (2020) ^	CALY-002 (1)	Anti-IL-15 mAb	No
IgE	Potential role in food allergy-mediated EoE	1	→ NCT00123630 (2005) #	Omalizumab	Anti-IgE mAb	No
TNFα	Pro-inflammatory cytokine (not Th2-specific)	1	→ NCT00523354 (2007) #	Infliximab (1)	Anti-TNFα mAb	No
Cpn60.1	Heat shock protein inhibiting leukocyte chemotaxis and diapedesis Inhibiting eosinophil recruitment in “in vitro” models	1	→ NCT05084963 (2021) ^	IRL201104 (1)	Cpn60.1 peptide	No
S1P	Integrity of endothelial barrier Leukocyte trafficking	1	→ NCT04682639 (2021) ^	Etrasimod (1)	S1P-receptor modulator	No

CRTH2: chemoattractant receptor-homologous molecule-positive Th2 cells; EoE: eosinophilic esophagitis; ILC2s: type 2 innate lymphoid cells; IL-4: Interleukin-4; IL-5: Interleukin-5; IL-13: Interleukin-13; IL-15: Interleukin-15; IL-4Rα: IL-4 receptor alpha; Ig: immunoglobulin; MCs: mast cells; mAb: monoclonal antibody; Siglec-8: sialic acid-binding immunoglobulin-like lectin 8; TSLP: thymic stromal lymphopoietin; * not yet recruiting; ^ undergoing recruitment; # published; $ early termination.

## Data Availability

No new data were generated or analyzed in support of this research.

## Abbreviations

Eosinophilic esophagitis (EoE), T helper 2 (Th2), High-Power Field (HPF), dilated intercellular spaces (DISs), basal zone hyperplasia (BZH), surface epithelial alterations (SEAs), proton pump inhibitors (PPIs), swallowed topical corticosteroids (STCs), atopic dermatitis (AD), allergic rhinitis (AR), chronic rhinosinusitis with nasal polyps (CRSwNP), epithelial cells (ECs), dendritic cells (DCs), Interleukin (IL), thymic stromal lymphopoietin (TSLP), type 2 innate lymphoid cells (ILC2s), chemoattractant receptor-homologous molecule-positive Th2 cells (CRTH2), mast cells (MCs), immunoglobulin (Ig), Oral Immuno-Therapy (OIT), sialic acid-binding immunoglobulin-like lectin (Siglec), Filaggrin (FLG), danger-associated molecular patterns (DAMPs), Toll-Like Receptors (TLRs), Respiratory Syncytial Virus (RSV), Pathogen-Associated Molecular Patterns (PAMPs), Genome-Wide Association Study (GWAS), single nucleotide polymorphisms (SNPs), epidermal differentiation complex (EDC), monoclonal antibodies (mAbs), Transforming Growth Factor (TGFβ), Calpain 14 (CAPN14), Desmoglein-1 (DSG-1), Desmoplakin (DSP), Food Elimination Diets (FEDs), eosinophil-derived neurotoxin (EDN), eosinophil peroxidase (EPO), eosinophil cationic protein (ECP), major basic protein (MBP), IL-4 receptor alpha subunit (IL-4Rα), Food and Drug Administration (FDA), European Medicines Agency (EMA), Randomized Controlled Trial (RCT), alpha subunit of IL-5 receptor (IL5Rα), Eosinophilic Gastritis (EG), Eosinophilic Duodenitis (ED), Natural Killer (NK), Tumor Necrosis Factor alpha (TNFα).
